# Gender effects of the COVID-19 pandemic in the Swiss labor market

**DOI:** 10.1186/s41937-022-00099-z

**Published:** 2022-12-08

**Authors:** Corinne Dubois, Luisa Lambertini, Yu Wu

**Affiliations:** 1 Department of Economics, University of Fribourg, Fribourg, Switzerland; 2 Chair of International Finance, École polytechnique fédérale de Lausanne (EPFL), Lausanne, Switzerland

**Keywords:** COVID-19, Labor market inequality, Labor market policies, Gender gaps, E24, J01, J08, J21

## Abstract

We study the impact of the pandemic on gender gaps in labor market outcomes in Switzerland. Using the Swiss Labor Force Survey data, we document a significant increase in the gender gap in labor market participation. We find no evidence of a worsening of the unemployment gender gap during the pandemic, but we find that women were more likely to uptake short-time work (STW). Unlike the USA, the presence of children in the household did not worsen labor gender gaps. Sector and occupation, however, play an important role in explaining gender gaps. In particular, we document substantial heterogeneity in the effect of the pandemic on participation, STW, hours worked, and wage outcomes depending on the availability of telework in the respondent’s occupation.

## Introduction

The COVID-19 pandemic has deeply affected labor markets around the world. Previous studies [Alon et al. (2020), Collins et al. (2021), Bluedorn et al. (2021) among others] document that gender inequality in the labor market has increased during the COVID-19 crisis. For this reason, the term “she-cession” has been used by researchers and the media to refer to the coronavirus-induced recession started in 2020. Our paper is inspired by these novel findings on the gendered consequences of the COVID-19 pandemic in the labor market. We aim to understand if and how the COVID-19 pandemic affected gender gaps in the Swiss labor market. Switzerland is characterized by both high female participation rate and high female part-time employment rate. Comparing the gendered impact of COVID-19 in Switzerland with other countries may shed light on the role played by these two features in the response to lockdowns.

We start by documenting the gender gap in labor market outcomes in terms of indicators such as labor market participation, unemployment, hours worked, leave of absence, and recourse to short-time work (STW), a public program that covers employees’ salaries when a company reduces operation due to reasons outside its control.^1^ We then examine which gaps have changed and how during the COVID-19 pandemic. Our empirical analysis uses Swiss Labor Force Survey (SLFS) data. It relies on a diff-in-diff approach after controlling for usual labor market confounders, including age, education, location, sector, and occupation. We consider the pandemic as a natural experiment and assume that the lockdown measures were strictly exogenous to the labor market situation. Under strict exogeneity, this approach identifies the pandemic’s causal effects on the labor market. We assume that stricter lockdown measures have a stronger impact on the labor market and use a continuous variable, a lockdown stringency index, to capture the “normalized” effect by unit of stringency.

The COVID-19 pandemic reduced employment and participation, and it increased unemployment for male as well as female respondents in Switzerland. However, there was an additional negative effect on women’s labor market participation (extensive margin), which is in line with findings in other countries (Bluedorn et al. 2021). Specifically, the transition from unemployment to inactivity increased more for women than men upon impact, and the probability of remaining non-active during the second phase of the pandemic was also higher for women. We document that while the unemployment gender gap did not worsen during the pandemic, women have been more likely to be on STW than men. We also find that the gender gap in working hours (intensive margin) was not affected by the pandemic.

We then analyze how family characteristics influence the evolution of the gender gaps during COVID-19. We find that being married or having children is associated with lower rates of inactivity and STW participation for women during the pandemic. The result pertaining to the presence of children contrasts with the effects documented in other countries. For example, Fabrizio et al. (2021), and Zamarro and Prados (2021) report a disproportionately negative impact of the crisis on mothers in the USA due to school closures; Andrew et al. (2020) find a similar effect in the UK. Several factors contribute to the opposite effect we find for Switzerland. First, the large recourse to STW allowed women to keep their employment contracts with a reduced workload, thus allowing them to care for children. Second, 50% of employed women have a part-time job in Switzerland. We believe that part-time work made it easier to take care of children during the pandemic, especially if the partner was working from home. Third, school closures in Switzerland were considerably shorter than in the UK or the USA, allowing mothers a swift return to work. Finally, the result could be driven by a family insurance mechanism, where women keep or take new employment or increase their working hours to compensate the income loss of their partners.

Next, we turn our attention to the role of occupation and telework availability in explaining the gender gap dynamics during the pandemic. After controlling for COVID-19 occupation- and sector-specific effects, we find that the non-active gender gap disappears, suggesting that the differential impact is mainly driven by the fact that women were more likely to work in sectors and occupations hit hard by the pandemic. When we remove COVID-19 sector and occupation fixed effects and categorize occupations by telework availability, we find that the non-active gender gap disappears for respondents with high-teleworkable jobs. In contrast, the gap persists among respondents with low-teleworkable jobs. This result is in line with the findings in Alon et al. (2022) and Shibata (2021), who argue that the increase of telework availability reduces gender inequalities in the labor market. We also find that gender gaps in hours worked and uptake of STW widened in low-teleworkable occupations, leading to a higher probability for women in these occupations to experience an income reduction during the pandemic.

The rest of the paper is organized as follows. Section 2 reviews the relevant literature. Sections 3 and 4 describe the labor force survey data and our regression design. Section 5 presents changes in labor market status. Section 6 discusses the reliance on STW scheme. Section 7 focuses on employed respondents and shows how they adjusted their working time. Section 8 shows changes in gender wage gap. Section 9 concludes.

## Literature

Our work relates to the growing literature analyzing the labor market impact of the COVID-19 pandemic. Cajner et al. (2020a), Bick and Blandin (2021), Coibion et al. (2020), Forsythe et al. (2020), Juranek et al. (2021) and Gupta et al. (2020) provide empirical evidence that this pandemic has resulted in large employment losses and substantial declines in hours worked. A subset of this literature focuses on the heterogeneous effects of the COVID-19 crisis across sectors, occupations and worker characteristics [e.g., Leibovici et al. (2020), Mongey et al. (2021), Montenovo et al. (2020), Cajner et al. (2020) and Benzeval et al. (2020)]. Our analysis confirms a large negative effect of the COVID-19 crisis on the Swiss labor market, but it rather focuses on the differential impact for male and female workers. Since Switzerland is characterized by both a high female participation rate and a high female part-time employment rate, it is interesting to investigate the labor market gender effects of the COVID-19 pandemic and compare it with other countries. If part-time jobs allow women to better balance work and family needs, we may expect the impact of the pandemic on gender gaps in labor market outcomes to be smaller in Switzerland relative to the other countries. Our results indicate an increased gender gap in non-participation during COVID-19, which is broadly in line with recent work on the disproportionately negative impact of the pandemic on women (Zamarro et al. 2020; Alon et al. 2020; Couch and Fairlie 2020; Albanesi and Kim 2021). Unlike previous studies carried out on the USA, German, and Spanish data (Stantcheva 2022), we observe no significant change in the gender gap of being unemployed and in hours worked during the pandemic in Switzerland.

An important channel for the stronger effects on women’s labor market outcomes relative to male counterparts is that women bear the brunt of increased childcare needs due to school and daycare closures, which lead them to reduce hours worked or exit the labor market altogether (Del Boca et al. 2020; Queisser et al. 2020; Alon et al. 2020; Collins et al. 2021; Farré et al. 2020; Sevilla and Smith 2020). Our results for Switzerland are different from most previous findings, as we find that motherhood did not contribute to increase the gender gap during the COVID-19 crisis. A high female part-time employment rate in Switzerland may give women more flexibility to hold on to their jobs or work more to compensate for their partner’s income loss.

Another channel documented in the literature is that women may be over-represented in the most affected sectors and occupations. Mongey et al. (2021) suggest that social distancing rules had the biggest effect on female-dominated sectors, namely the service industry. Alon et al. (2022) provide a decomposition analysis and show that the differential occupation distribution accounts for 12 percent of the gender gap in the employment decline. Our results are consistent with these findings. We document that gender differences in the distribution across occupations and sectors explains the gender gap in labor market participation during COVID-19. We also show the importance of telework availability on gender gaps during the pandemic. In line with previous findings by Albanesi and Kim (2021), Shibata (2021) and Mongey et al. (2021), we show that the possibility to work from home greatly reduced (or even eliminated) gender gaps in the concerned occupations.

Finally, this paper touches upon the literature on the effectiveness of labor market policies in cushioning the economic consequences of COVID-19. During this crisis, a prominent feature in policy has been the introduction or expansion of furloughing and STW schemes. Kopp and Siegenthaler (2017), Hijzen and Venn (2011) and Abraham and Houseman (2014) find that STW helped stabilizing employment during the Great Recession. Adams-Prassl et al. (2020) compare the impact of the COVID-19 crisis in the UK, the USA, and Germany. They show that German employees were less affected by the crisis thanks to a well-established STW scheme. Our contribution to this literature is to study the gender effects of STW policies. In line with other studies, we find that STW played a significant role in Switzerland during the crisis; our innovative finding is that women have been more likely than men to use it.

## Data and descriptive statistics

The Swiss Labor Force Survey (SLFS) is a quarterly survey conducted in Switzerland since 1991 among residents aged 15 and older. It aims to provide information on the labor force structure and labor market patterns. The survey is carried out by telephone on a representative sample of the population (around 120’000 annual interviews). The SLFS sample is a 4-wave rotating panel: The persons who participate in the survey are interviewed four times over 15 months.^2^ The SLFS includes questions on current and previous labor market status, working conditions, occupation, salary, job seeking, as well as general questions on education, household composition, and other demographic characteristics. Our dataset includes quarterly data for the period 2019Q1 to 2020Q4; it contains a total of 231’667 observations, i.e., approximately 30’000 observations per quarter.

**Fig. 1:**
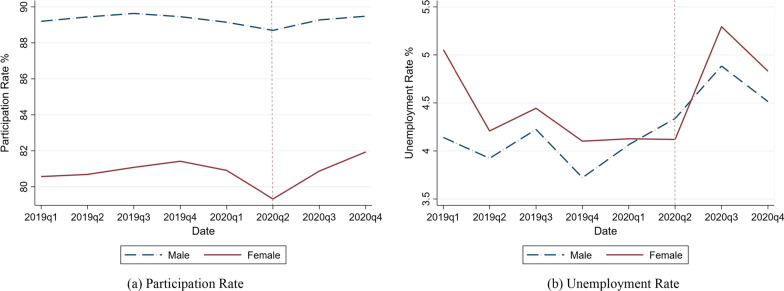
Labor market status by gender. *Notes* The participation rate is measured as the number of working-age respondents in the labor force as a percentage of the total number of working-age respondents. The unemployment rate is calculated as the number of working-age respondents currently unemployed as a percentage of the total number of working-age respondents in the labor force. We break down these indicators by gender, measured as a percentage of each gender group

Figure 1 reports the dynamics of labor market status by gender between 2019Q1 and 2020Q4, and it reveals that the pandemic affected men and women differently. Female labor market participation fell sharply in the second quarter of 2020 and returned to its pre-pandemic level by the end of the year. In contrast, the reduction in male labor market participation was less severe. Interestingly, the female unemployment rate did not increase in 2020Q2, but it jumped by 1.2 percentage points in 2020Q3; on the other hand, the male unemployment rate increased continuously over the two quarters. This evidence suggests that women were more likely than men to drop out of the labor market at the height of the pandemic. This is not due to the difference in the probability of getting COVID-19 across genders. Women appear slightly more likely to get the virus or more likely to test if they are positive, but the difference is very small.

**Fig. 2:**
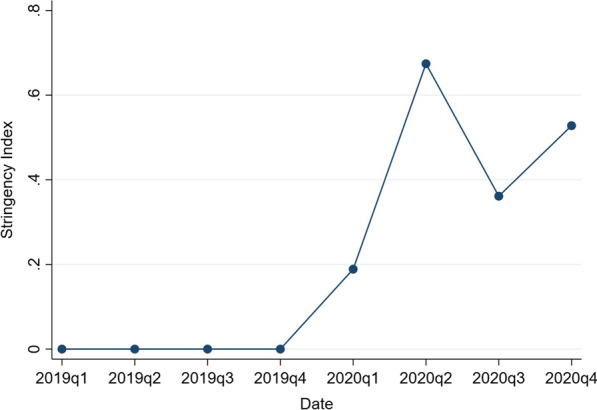
KOF stringency index. *Notes* This index record the stringency of COVID-19 policy measures in Switzerland. It is constructed as the (population) weighted average of the cantonal indexes. The values range from 0 (=no measures) to 1 (=full lockdown). *Source* KOF Swiss Economic Institute

We use the KOF stringency index,^3^ to capture the stringency of lockdown measures during the COVID-19 pandemic. The index is available at the cantonal level daily; we construct the national index as the (population) weighted average of the cantonal indices and convert daily to quarterly values by averaging.^4^ The value of the index ranges from 0 (= no measures) to 100 (= full lockdown). In our analysis, we use the national KOF stringency index normalized to a scale from 0 to 1, which is displayed in Fig. 2. Stringency measures peaked in the second quarter of 2020, reaching the value of 0.75. Toward the end of February 2020, the Swiss Federal Council banned all events with more than 1,000 participants; in March, most shops were closed nationwide and all public gatherings of more than 5 people were banned. Lockdown measures were eased in the third quarter and raised again in the fourth quarter of 2020. In October 2020, as cases surged again, the Federal Council limited public gatherings to a maximum of 15 people and made masks mandatory in all enclosed public spaces.

**Fig. 3:**
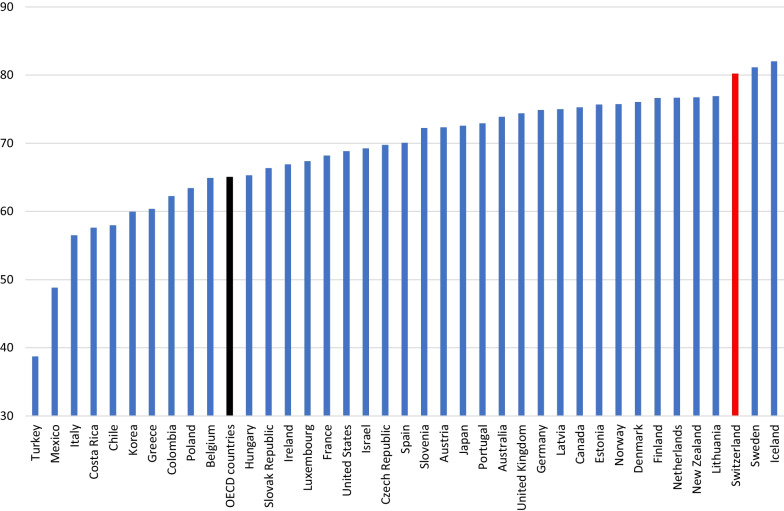
Female labor market participation (2019). *Notes* Data from the OECD labor force statistics. The labor force participation rates are calculated as the labor force divided by the total working-age population (15–64)

**Fig. 4:**
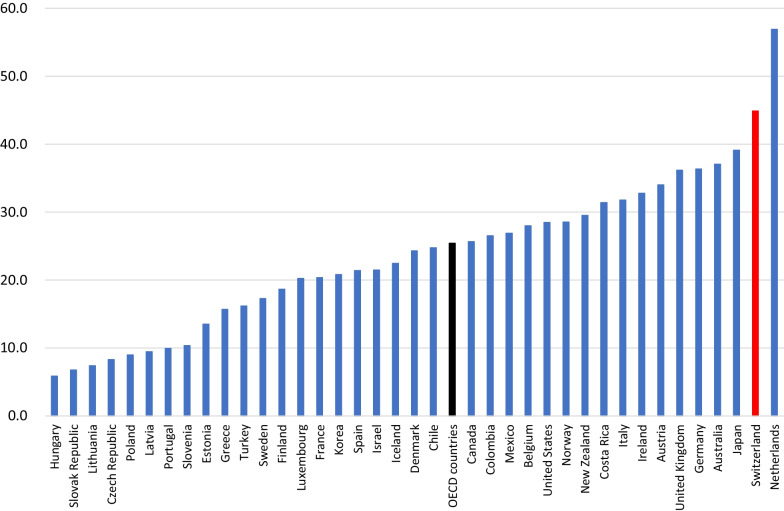
Women employed in part-time work, as percent of total employed women (2019). *Notes* Data (for all countries except the USA) from the OECD labor force statistics; data for the USA come from the Current Population Survey. The part-time rate is calculated as the number of employed women working less than 30 hours (35 for the USA) weekly over the total number of employed women.

Figure 3 reports the female labor market participation rate in Switzerland and other OECD countries. It shows that the Swiss female participation rate is the third highest of all OECD economies and about 15% points above the OECD average. However, a distinctive feature of the Swiss labor market is the widespread use of part-time work, especially among women. The percentage of employees (both male and female) in a part-time job in 2019 was on average 16.7% in OECD countries, but 26.9% in Switzerland. Figure 4 shows that the percentage of part-time workers among women employees in Switzerland is particularly high (44.9%) and the second largest among all OECD economies. These facts suggest that women in Switzerland benefit from more flexibility in their employment relative to other countries. The possibility to work part-time allows work-family balance and encourages participation to the labor market of women and, especially, of mothers. In our sample, 35% of employed women without children work part-time, while 65% of mothers of children between 0 and 6 and 63% of mothers of 7–14-year-old children work part-time. These distinctive features of the Swiss labor market play an important role in the response of women to the COVID-19 crisis and make Switzerland an interesting case study to analyze.

## Regression design

We use a diff-in-diff specification to study whether COVID-19 impacted differently men and women on the labor market. The typical regression specification looks as follows:

1 $$\begin{aligned} y_{i,t}&=\alpha + \gamma _1 {\hbox {female}}_{i} + \gamma _2 {\hbox {CovInd}}_t \nonumber \\&\quad + \gamma _\mathrm{{cov}} {\hbox {female}}_{i} \times {\hbox {CovInd}}_t + \gamma _3 X_{i,t} + \epsilon _{i,t}, \end{aligned}$$

where $y_{i,t}$ is the dependent variable of interest, including labor market status, STW, searching for jobs, having worked last week, hours worked, and taking family leave. $female_{i}$ is a binary variable equal to one if the respondent is female. $CovInd_t$ is the COVID-19 stringency index shown in Fig. 2;^5^$X_{i,t}$ is a vector of covariates, including age cohort, indicators for occupation, location, sector of economic activity, and the level of education. Suppose $y_{i,t}$ is the labor market status of the respondent, which is a dummy equal to one for employed and zero for unemployed and non-active; then, $\gamma _1$ measures the differential likelihood of female respondents being employed relative to male respondents and $\gamma _2$ is the differential likelihood of both male and female respondents being employed during the COVID-19 crisis relative to normal times; $\gamma _\mathrm{{cov}}$ is our parameter of interest and it captures the employment gender gap differential as the stringency of COVID-19 lockdown measures change.^6^

To capture the effect of a specific factor $z_i$ on female’s differential likelihood of being employed/unemployed/on STW/etc., during COVID-19, we use a triple-diff regression of the following type:

2 $$\begin{aligned} y_{i,t}&= \alpha + \gamma _1 {\hbox {female}}_{i} + \gamma _2 {\hbox {CovInd}}_t + \gamma _3 z_{i,t} \nonumber \\&\quad +\gamma _{4} {\hbox {female}}_{i} \times z_{i,t} + \gamma _{5} {\hbox {CovInd}}_t \times z_{i,t} \nonumber \\&\quad + \gamma _\mathrm{{cov}} {\hbox {female}}_{i} \times {\hbox {CovInd}}_t \nonumber \\&\quad +\gamma _{\mathrm{{cov}},z} {\hbox {female}}_{i} \times {\hbox {CovInd}}_t \times z_{i,t} +\gamma _{6} X_{i,t} + \epsilon _{i,t}, \end{aligned}$$

where $z_{i,t}$ is the specific independent variable of interest, including civil status, presence of children in the household, or telework availability. $\gamma _\mathrm{{cov}}$ captures the gender gap differential as the stringency index changes among those respondents who do not share factor $z_i$. $\gamma _{\mathrm{{cov}},z}$ captures the differential gender gap for respondents with and without factor $z_i$ during the COVID-19 pandemic.

## COVID-19 and labor market status

We start our empirical analysis by studying how the COVID-19 pandemic impacted the labor market status of male and female respondents and which characteristics explain differential effects.

### Effect of gender and COVID-19 on labor market status

Table 1 presents the estimates based on regression (1) with labor market status as the dependent variable. Labor market status is set as a dummy with value 1 if the person is employed (column 1), unemployed (column 2), or non-active (column 3); we control for the respondent’s age, level of education, canton of residence, type of occupation (ISCO code), and the type of economic activity as measured by NOGA 1st level code.^7^ In this regression, we only consider working-age population, i.e., respondents between age 15 and 64.

**Table 1:** COVID-19 and labor market status

	Employed	Unemployed	Non-active
Female	−0.0408$$^{***}$$	−0.00166	0.0412$$^{***}$$
	(0.00232)	(0.00139)	(0.00205)
CovInd	−0.0165$$^{***}$$	0.00556$$^{***}$$	0.0117$$^{***}$$
	(0.00278)	(0.00211)	(0.00252)
Female $\times$ CovInd	−0.00165	−0.00461	0.00583$^{*}$
	(0.00385)	(0.00292)	(0.00350)
Constant	0.727$$^{***}$$	0.0291	0.243$$^{***}$$
	(0.0396)	(0.0233)	(0.0350)
Age FE	YES	YES	YES
Canton FE	YES	YES	YES
Education FE	YES	YES	YES
NOGA FE	YES	YES	YES
ISCO FE	YES	YES	YES
Observations	186881	186881	186881
$R^2$	0.417	0.0423	0.459

Estimates from regression (1) of labor market status on a constant, female dummy (1 for women and 0 otherwise), COVID-19 stringency index and its intersection with the female dummy. Sample includes respondents aged 15 to 64. Regressions estimated with linear probability model, including random effects. Robust standard errors in parentheses

*p < 0.1, **p < 0.05, ***p < 0.01

Several findings in Table 1 are worth mentioning. First, women are less likely to be employed and more likely to be non-active than men in normal times; this confirms the well-known lower female participation presented in Fig. 1. Second, the COVID-19 pandemic has decreased the likelihood of employment and increased that of being unemployed and non-active for male as well as female respondents. Third, women are more likely than men to be non-active during COVID-19.^8^ Since we do not control for the respondent’s previous labor market status,^9^ this suggests that women either became more likely to exit the labor market or became less likely to reenter it during the pandemic. Fourth, lockdown measures did not make women more likely to be unemployed than men.

**Fig. 5:**
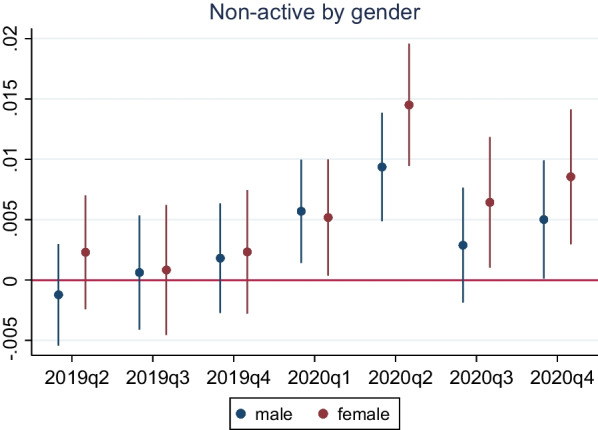
Non-active Over Time for Men and Women. *Notes* Estimates from regression (3) of non-active dummy on quarterly dummies, run separately for men (blue) and women (red), with 95% confidence intervals. Regressions estimated with linear probability model, including random effects.

To analyze how the probability of being non-active evolved over time for men and women, we run the following regression:

3 $$\begin{aligned} y_{i,t} = \alpha + \sum _{s=2019Q2}^{2020Q4}\gamma _{\mathrm{{cov}},s} Q_{s,t} +\gamma _2 X_{i,t} + \epsilon _{i,t}, \end{aligned}$$

where $y_{i,t}$ is the dummy for being non-active, $Q_{s,t}$ are quarterly dummies, and $X_{i,t}$ is the same vector of covariates as in regression (1). The coefficients of interest, $\gamma _{\mathrm{{cov}},s}$, indicate the differential propensity of being non-active in quarter *s* compared to 2019Q1. The regression is run separately for male and female respondents, and the coefficient estimates of the quarterly dummies are plotted in Fig. 5. First, we observe that the probability of being non-active increased during COVID-19; it peaked in 2020Q2, when the strictest lockdown measures were adopted; it fell in 2020Q3, when sanitary standards were relaxed; it increased again in quarter 4, following the resurgence of the pandemic in the Fall. Second, non-active propensities were systematically higher, although not significantly, for women. For example, the probability of being non-active in 2020Q2, relative to 2019Q1, increased by 0.9 percentage points for men but 1.5 percentage points for women.

### Labor market transitions by gender

**Table 2:** Labor market transition probabilities

		Men				Women		
		E	U	NA		E	U	NA
(a). Changes in 2020Q2	L.E	– 0.18	0.22	– 0.04	L.E	– 0.29	0.07	0.23
	L.U	– 10.62	6.68	3.94	L.U	– 12.09	6.28	5.81
	L.NA	– 0.32	– 0.84	1.17	L.NA	– 1.23	– 0.1	1.32

(b). Changes in 2020Q3–Q4	L.E	0.17	0.16	– 0.32	L.E	0.2	0.05	– 0.24
	L.U	– 6.94	12.36	– 5.42	L.U	1.44	6.09	– 7.52
	L.NA	1.74	1.64	– 3.38	L.NA	– 1.46	0.31	1.15

L.E, L.U, L.NA show previous statuses

Sample includes respondents aged 15 to 64 for which we have information on employment status in two consecutive quarters. Panels (a) and (b) report the changes in probabilities of status transition from one quarter to the next in 2020Q2 and 2020Q3–Q4 (average of the two quarters), respectively, relative to the year before (pre-COVID times). Results are reported in percentage points

*E* employed, *U* unemployed, *NA* non-active

To better understand the effect of COVID-19 on labor market gender gaps, we calculate the average transition probabilities between different labor market statuses from one quarter to the next separately for male and female respondents over time. Table 2 panels (a) and (b) report the changes in 2020Q2 and 2020Q3-Q4, respectively, relative to the year before, when no lockdown restrictions were in place. This is to say, we compare the transition probabilities in 2020Q2 to 2019Q2, and in the average of 2020Q3-Q4 to the average of 2019Q3-Q4, respectively, so that we can control for seasonal factors and assess how transition probabilities were affected by COVID-19. *E*, *U*, and *NA* refer to respondents’ current labor market status (employed, unemployed, and non-active, respectively); *L.E*, *L.U*, and *L.NA* indicate their status in the previous quarter.

Panel (a) reveals how transition probabilities changed in 2020Q2, when the strictest lockdown measures were in place. Interestingly, previously employed male and female respondents were little affected by the pandemic; this result is likely the consequence of the government policies to protect employment, such as STW and liquidity provision to firms.^10^ Still, there are gendered differences among previously employed respondents: Women became less likely than men to transition to unemployment and more likely to transition to non-activity. Among previously unemployed respondents, we see a decrease in the probability of finding employment accompanied by an increase in the likelihood of remaining unemployed or becoming non-active, with the latter effect being much larger for women. Finally, among previously non-active respondents, we observe a worsened gender gap as women became less likely than men to transition to employment. These results confirm the disproportionate effect of the pandemic on women documented in Table 1.

Panel (b) reports how transition probabilities changed in the period of 2020Q3-Q4 relative to 2019Q3-Q4.^11^ Lockdown measures were relaxed in quarter 3 and tightened again in quarter 4 of 2020; nevertheless, measures were at all times milder than in 2020Q2. Panel (b) shows that, again, previously employed respondents did not experience substantial changes in their labor market status. There are signs of labor market recovery in the second half of 2020, as previously unemployed respondents are more likely to remain unemployed rather than exit the labor market, and previously non-active respondents become more likely to reenter the labor force. However, the latter effect is much weaker for women than for men, as women were still more likely to remain non-active.

To summarize, women were more likely to leave the labor market at the peak of the pandemic and not to reenter as the labor market started to recover.

### Labor market status and marital status

This section studies if family-related characteristics explain the evolution of the gender gaps in the labor market during the pandemic. We start by studying the role of marital status using regression (2). The dependent variables are still the labor market status dummies (*employed*, *unemployed*, and *non-active*); we add to the explanatory variables of Table 1 the marital status dummy (1 for married or in a registered relationship and 0 otherwise), its interactions with the female dummy and the COVID-19 index, as well as the triple interaction of female, COVID-19 index, and marital status.

**Table 3:** COVID-19, labor market, and marital status

	Employed	Unemployed	Non-active
Female	−0.00362	−0.00551$$^{***}$$	0.00864$$^{***}$$
	(0.00325)	(0.00197)	(0.00288)
CovInd	−0.0157$$^{***}$$	0.00737$$^{**}$$	0.0103$$^{***}$$
	(0.00413)	(0.00313)	(0.00375)
Female $$\times$$ CovInd	−0.0115$$^{**}$$	−0.00586	0.0162$$^{***}$$
	(0.00576)	(0.00437)	(0.00523)
Married	0.0311$$^{***}$$	−0.0134$$^{***}$$	−0.0166$$^{***}$$
	(0.00323)	(0.00196)	(0.00286)
Married $$\times$$ female	−0.0692$$^{***}$$	0.00732$$^{***}$$	0.0603$$^{***}$$
	(0.00559)	(0.00424)	(0.00508)
Married $\times$ CovInd	−0.00110	−0.00373	0.00225
	(0.00559)	(0.00424)	(0.00508)
Married $$\times$$ female $$\times$$ CovInd	0.0176$$^{**}$$	0.00256	−0.0185$$^{***}$$
	(0.00776)	(0.00589)	(0.00704)
Constant	0.706$$^{***}$$	0.0329	0.260$$^{***}$$
	(0.0395)	(0.0234)	(0.0350)
Age FE	YES	YES	YES
Canton FE	YES	YES	YES
Education FE	YES	YES	YES
NOGA FE	YES	YES	YES
ISCO FE	YES	YES	YES
Observations	186881	186881	186881
$R^2$	0.419	0.0429	0.460

Estimates from regression (1) of labor market status on a constant, female dummy (1 for women and 0 otherwise), COVID-19 stringency index, marital status dummy (1 for married/in a registered relation and 0 otherwise), and their interactions. Sample includes respondents aged 15 to 64. Regressions estimated with linear probability model, including random effects. Robust standard errors in parentheses

*p < 0.1, **p < 0.05, ***p < 0.01

Table 3 reports the estimates. In normal times, marriage worsens all gender gaps: married women are less likely to be employed, more likely to be unemployed or non-active than married men. Interestingly, however, marriage does not contribute to the increased gender gaps during the pandemic. In other words, the COVID-19 pandemic increased gaps between unmarried men and women but did not change them for married respondents.^12^ A possible explanation for the negligible effect of marriage is family insurance. During the COVID-19 pandemic, married women were more likely to become or remain employed in order to offset an income reduction experienced by their partner.

### Labor market status and child care

In this section, we study how the presence of children in the household affects the differential impact of the COVID-19 pandemic using regression (2). We add to our starting set of explanatory variables a dummy *child0-6*, which equals 1 if the respondent has children 6 years old or younger, and a dummy *child7-14*, which is equal to 1 if the respondent has children between 7 and 14 years old;^13^ we also include the interaction of our two children dummies with the female dummy, their interaction with the COVID-19 index, and the triple interaction of female, COVID-19 index, and children dummies.

**Table 4:** COVID-19, labor market status, and child care responsibility

	Employed	Unemployed	Non-active
Female	−0.0173$$^{***}$$	−0.00726$$^{***}$$	0.0230$$^{***}$$
	(0.00276)	(0.00167)	(0.00244)
CovInd	−0.0167$$^{***}$$	0.00449$$^{*}$$	0.0129$$^{***}$$
	(0.00335)	(0.00254)	(0.00304)
Female $$\times$$ CovInd	−0.00990$$^{**}$$	−0.00409	0.0135$$^{***}$$
	(0.00466)	(0.00354)	(0.00423)
Child0-6	0.0309$$^{***}$$	−0.0153$$^{***}$$	−0.0165$$^{***}$$
	(0.00448)	(0.00279)	(0.00398)
Child7-14	0.0353$$^{***}$$	−0.0162$$^{***}$$	−0.0191$$^{***}$$
	(0.00457)	(0.00288)	(0.00407)
Child0-6 $$\times$$ female	−0.110$$^{***}$$	0.0233$$^{***}$$	0.0881$$^{***}$$
	(0.00604)	(0.00376)	(0.00537)
Child7-14 $$\times$$ female	−0.0429$$^{***}$$	0.0142$$^{***}$$	0.0294$$^{***}$$
	(0.00612)	(0.00384)	(0.00544)
Child0-6 $\times$ CovInd	−0.00218	0.00586	−0.00375
	(0.00821)	(0.00622)	(0.00745)
Child7-14 $\times$ CovInd	0.00714	0.000584	−0.00723
	(0.00841)	(0.00638)	(0.00763)
Child0-6 $$\times$$ female $$\times$$ CovInd	0.0453$$^{***}$$	−0.00794	−0.0343$$^{***}$$
	(0.0112)	(0.00853)	(0.0102)
Child7-14 $\times$ female $\times$ CovInd	−0.000995	0.00734	−0.0101
	(0.0115)	(0.00874)	(0.0104)
Constant	0.714$$^{***}$$	0.0384	0.247$$^{***}$$
	(0.0410)	(0.0245)	(0.0363)
Age FE	YES	YES	YES
Canton FE	YES	YES	YES
Education FE	YES	YES	YES
NOGA FE	YES	YES	YES
ISCO FE	YES	YES	YES
Observations	175360	175360	175360
$R^2$	0.422	0.0426	0.462

Estimates from regression (2) of labor market status and no job search for family reasons on a constant, female dummy (1 for women and 0 otherwise), COVID-19 stringency index, child dummies [child0-6 for having child(ren) under 7 years old, child7–14 for having school age child(ren)], and their interactions. Sample includes respondents aged 15 to 64. Regressions estimated with linear probability model, including random effects. Robust standard errors in parentheses. The sample is restricted to respondents who share complete information on family structure

*p<0.1, **p<0.05, ***p<0.01

Table 4 displays the estimates. In normal times, women are overall less likely to be employed and more likely to be non-active than men; the presence of children increases these disparities, especially when children are under 7 years old. During COVID-19, however, the presence of children does not contribute to a worsening of gender gaps. In particular, the gender gap in employment and non-participation worsened among respondents without children. Still, these gaps between fathers and mothers of school-aged children (7 to 14) did not change and even significantly diminished for mothers of young children during the pandemic.^14^ In other words, the presence of young children actually increases the probability of employment and decreases that of non-participation for women during COVID-19.

These findings differ from previous studies for other countries, such as the UK and the USA, which found that the gender gap in employment worsened with the presence of children during the COVID-19 crisis. There are several factors at play in reducing the gap. First, 33% of the Swiss labor force was put in STW during the lockdown (see Sect. 6). As a result, women kept their employment but with a reduced workload and were thus able to reconcile family responsibility and employment during the crisis. Second, school/kindergarten closure policies were relatively lenient in Switzerland.^15^ Third, since mothers in Switzerland usually work part-time, they could more easily hold on to their job while meeting higher childcare needs during the crisis. Fourth, men in STW or working from home helped share child care responsibilities during the lockdown.

### Labor market status, sector and occupation effects

The COVID-19 pandemic affected sectors and occupations differently. Sectors such as food services and accommodation or entertainment were hit hard, while others such as information technology or financial services remained largely unscathed. Table 5 re-estimates regression (2) allowing for a differential effect of COVID-19 on each sector and occupation. This is to say that, we keep the labor market status dummies (*employed*, *unemployed*, and *non-active*) as dependent variables and add $occupation \times CovInd$ and $sector \times CovInd$ fixed effects. The coefficient estimate of the female and COVID-19 interaction for non-active respondents is one order of magnitude smaller than our estimates in Table 1 and statistically insignificant. This suggests that the observed gender gap in the non-active status is mainly due to women being predominantly employed in sectors and occupations that suffered more during the crisis.

**Table 5:** COVID-19, labor market status, and occupation/sector-specific effects

	Employed	Unemployed	Non-active
Female	−0.0410$$^{***}$$	−0.00251$$^{*}$$	0.0423$$^{***}$$
	(0.00236)	(0.00143)	(0.00209)
CovInd	0.0626	−0.0112	−0.0627
	(0.0958)	(0.0716)	(0.0869)
Female $\times$ CovInd	−0.000766	−0.000875	0.000807
	(0.00431)	(0.00327)	(0.00391)
Constant	0.712$$^{***}$$	0.0305	0.259$$^{***}$$
	(0.0436)	(0.0269)	(0.0387)
Age FE	YES	YES	YES
Canton FE	YES	YES	YES
Education FE	YES	YES	YES
NOGA FE	YES	YES	YES
ISCO FE	YES	YES	YES
NOGA $\times$ CovInd	YES	YES	YES
ISCO $\times$ CovInd	YES	YES	YES
Observations	186881	186881	186881
$R^2$	0.417	0.0428	0.459

We show estimates from regression (1) of labor market status after adding the interaction of the COVID-19 stringency index and dummies for occupations and sectors as controls. Regressions estimated with linear probability model, including random effects. Robust standard errors in parentheses

*p<0.1, **p<0.05, ***p<0.01

### Labor market status and telework availability

**Fig. 6:**
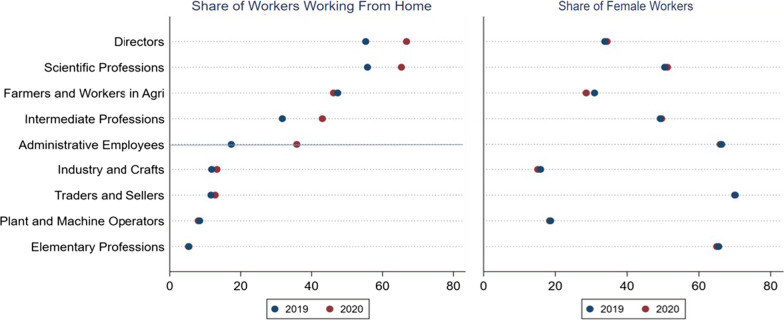
Telework Availability and Female Share by Occupation. *Notes* The share of workers working from home (telework availability) is measured as the number of respondents who worked from home in the last 4 weeks as a percentage of the total number of respondents in each occupation group. The share of female workers is calculated as the number of respondents who are female as a percentage of the total number of respondents in each occupation group. We rank occupations in a descending order of telework availability in 2020

We use regression (2) to test whether the availability of telework in a given occupation relates to the gender-specific effects of the COVID-19 pandemic. Telework availability is measured by the percentage of workers in a given occupation who worked from home occasionally or regularly during the last four weeks. We rank occupations by average telework availability in 2020, as displayed in Fig. 6. Telework availability varies greatly across occupations. In 2019, it ranged from approximately 5% for elementary professions to almost 60% for directors and scientific professions. Education and telework availability are positively correlated in our survey; the fraction of respondents, by educational level, who regularly or occasionally work remotely increases with the level of education—see Table 15 in Appendix. During the pandemic crisis of 2020, telework availability increased for the occupations that were already above median in 2019, but it remained almost unchanged for the other occupations. We construct a dummy variable *LowTele* that equals 1 for occupations that have below-median telework availability (industry and crafts, traders and sellers, plant and machine operators and elementary professions) and 0 for occupations with above-median telework availability (directors, scientific professions, farmers, intermediate professions, and administrative employees). Note that low-teleworkable occupations are typically blue-collar jobs requiring physical labor or services requiring physical presence; these occupations are not necessarily female-dominated, with the share of female workers ranging from 17 to 68%.

We add to the explanatory variables of Table 1 the telework dummy *LowTele*, its interactions with the female dummy and with the COVID-19 index, and the triple interaction $LowTele \times female \times CovInd$. Table 6 reports the estimates. Occupations with low teleworkability are characterized by lower employment, higher unemployment, and non-active rates for men and more so for women in normal times. Our results also confirm the findings of Table 1: The non-active gender gap has widened during the pandemic, but mainly so for occupations with low teleworkability. The estimated coefficient is indeed almost twice as large as the one estimated in Table 1. Hence, women have been more likely to exit the labor market in those professions where working from home is not possible.^16^

**Table 6:** COVID-19, labor market status, and telework availability

	Employed	Unemployed	Non-active
Female	−0.0413$$^{***}$$	0.00300$$^{*}$$	0.0370$$^{***}$$
	(0.00289)	(0.00164)	(0.00248)
CovInd	−0.0101$$^{***}$$	0.00346	0.00739$$^{**}$$
	(0.00354)	(0.00252)	(0.00299)
Female $\times$ CovInd	−0.00357	−0.000196	0.00336
	(0.00490)	(0.00350)	(0.00415)
LowTele	−0.0137$$^{***}$$	0.00803$$^{***}$$	0.00508$$^{*}$$
	(0.00358)	(0.00210)	(0.00306)
LowTele $$\times$$ female	−0.0176$$^{***}$$	−0.00170	0.0195$$^{***}$$
	(0.00479)	(0.00281)	(0.00410)
LowTele $$\times$$ CovInd	−0.0159$$^{**}$$	0.0117$$^{***}$$	0.00433
	(0.00623)	(0.00442)	(0.00527)
LowTele $$\times$$ female $$\times$$ CovInd	−0.00325	−0.0122$$^{*}$$	0.0163$$^{**}$$
	(0.00890)	(0.00631)	(0.00753)
Constant	0.883$$^{***}$$	0.0230$$^{***}$$	0.0942$$^{***}$$
	(0.00786)	(0.00445)	(0.00674)
Age FE	YES	YES	YES
Canton FE	YES	YES	YES
Education FE	YES	YES	YES
NOGA FE	YES	YES	YES
Observations	171218	171218	171218
$R^2$	0.0611	0.0787	0.0415

We show estimates from regression (2) of labor market status on a constant, female dummy (1 for women and 0 otherwise), COVID-19 stringency index, LowTele dummy (1 for respondents in an occupation with low telework availability and 0 otherwise), and their interactions. Regressions estimated with linear probability model, including random effects. Robust standard errors in parentheses. The sample is restricted to respondents who share complete information on occupation type

*$$p<0.1$$, **$$p<0.05$$, ***$$p<0.01$$

Putting our results together, we find that COVID-19 has worsened the non-active gender gap in specific occupations and, indirectly, via the sectoral impact of the pandemic. Children and marital status did not contribute to such effect.

## COVID-19 and short-time work

Our analysis this far has revealed a mild impact of the COVID-19 pandemic on unemployment in Switzerland, thanks to the massive uptake of STW. This section reviews STW policy in Switzerland and analyzes whether there was a gender gap in the recourse to STW during COVID-19.

### Short-time work in Switzerland

STW is a public policy that allows firms facing a fall in demand to keep their employees while transferring the cost to the government. The shortfall in demand must be outside the company’s control, and it may come, for example, from a downturn in economic activity, unusual weather conditions, or a pandemic. The STW compensation is paid to the employer and covers 80% of the loss of earnings attributable to the reduction in hours worked, up to a maximum insured gain of 148’200 CHF yearly. The aim is to reduce employees’ work without the need to lay them off. Note that employees have the right not to accept the STW compensation. In this case, the employer either continues paying the full salary or lays off the employee.

**Fig. 7:**
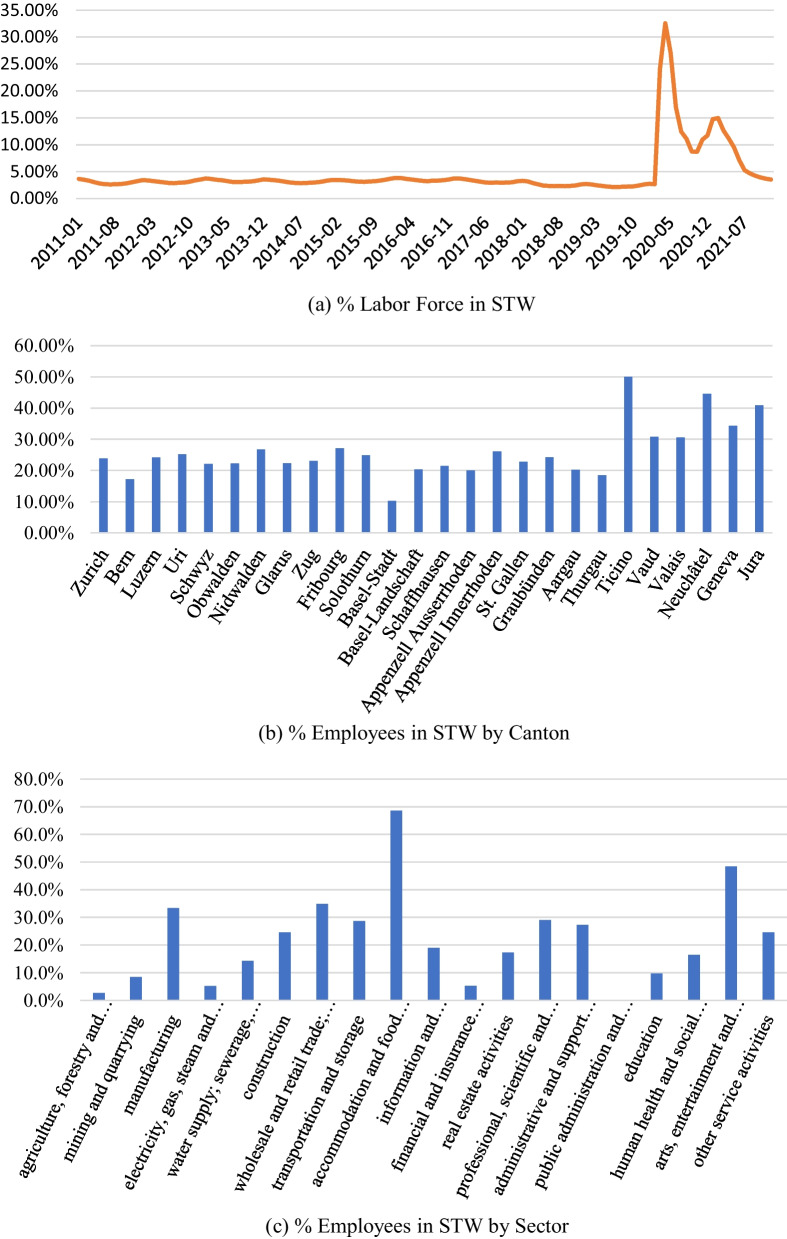
Employees in short-time work. *Notes* Data from the State Secretariat for Economic Affairs (SECO). In plots **b** and **c**, we show that number of employees in STW in April 2020, divided by the total number of employees in each canton/sector in 2019

In March 2020, the federal government decided to simplify and expedite the administrative procedures for requesting STW. The government reduced justification and reporting requirements, abolished the 2-day waiting period and the 10-day notice for requesting STW, and extended the maximum duration from 12 to 18 months until June 2021. Moreover, the government decided to broaden STW eligibility to apprentices and employees on fixed-term contracts. Panel (a) of Fig. 7 shows average STW rate as percentage of the Swiss labor force. The STW rate jumped to 29.3% in April 2020 and peaked again at 11.3% in February 2021.

Panel (b) of Fig. 7 reports the number of employees in STW in April 2020 as a percentage of the number of employed persons in that canton. We observe significant variation in the share of employees in STW across cantons, ranging from 10% for Basel city to 50% for Ticino. On average, the German-speaking cantons were less affected than the French and Italian-speaking cantons.

Finally, panel (c) of Fig. 7 plots the share of employees in STW by economic sector in April 2020. Lockdown measures included the complete shutdown of restaurants, non-essential shops, cinemas, theaters, etc.; hence, accommodation, food services, arts, and entertainment sectors were the most affected. Sectors dealing with essential goods and services, such as agriculture and electricity, were slightly impacted. Sectors where most of the work could be done remotely, such as the financial service sector, were barely affected.

### Effect of gender and COVID-19 on short-time work

**Table 7:** The effect of COVID-19 and gender on STW

	In short-time work		
	(1)	(2)	(3)
Female	0.000416	−0.000910	0.00154
	(0.00139)	(0.00172)	(0.00160)
CovInd	0.112$$^{***}$$	0.113$$^{***}$$	0.105$$^{***}$$
	(0.00251)	(0.00309)	(0.00304)
Female $$\times$$ CovInd	0.0311$$^{***}$$	0.0370$$^{***}$$	0.0198$$^{***}$$
	(0.00360)	(0.00443)	(0.00430)
Child0-6 $\times$ CovInd		0.00167	
		(0.00731)	
Child7-14 $\times$ CovInd		0.00130	
		(0.00752)	
Child0-6 $\times$ female $\times$ CovInd		−0.0204$^{*}$	
		(0.0105)	
Child7-14 $\times$ female $\times$ CovInd		−0.0177$^{*}$	
		(0.0106)	
LowTele			−0.000404
			(0.00207)
LowTele $\times$ female			−0.00146
			(0.00285)
LowTele $$\times$$ CovInd			0.0241$$^{***}$$
			(0.00540)
LowTele $$\times$$ female $$\times$$CovInd			0.0438$$^{***}$$
			(0.00787)
Constant	0.0146	0.0157	0.00246
	(0.0220)	(0.0238)	(0.00396)
Age FE	YES	YES	YES
Canton FE	YES	YES	YES
Education FE	YES	YES	YES
NOGA FE	YES	YES	YES
ISCO FE	YES	YES	
Observations	158250	148431	158162
$R^2$	0.0466	0.0480	0.0474

Estimates from regression (2) of STW dummy on a constant, female dummy, COVID-19 stringency index, child dummies (column 2), and low telework dummy (column 3). The sample is restricted to respondents who are employed or apprentices. Regressions estimated with linear probability model, including random effects. Standard errors in parentheses

*$$p<0.1$$, **$$p<0.05$$, ***$$p<0.01$$

This section analyzes whether women were more likely to be placed on STW during the COVID-19 crisis. Table 7 displays the estimates from regressions where the dependent variable is a dummy that equals 1 if the person is on STW and 0 otherwise. Column (1) includes the COVID-19 index, the female dummy, the interaction between the two, and controls for age, education, occupation type, sector of work, and canton of residence. It confirms that the COVID-19 pandemic increased STW for both men and women. The positive and significant coefficient on the interaction term $female \times CovInd$ indicates that women were more likely to be put on STW during the pandemic. Appendix Table 14 shows the estimates from a similar regression, but adding occupation times CovInd and sector times CovInd fixed effects. The coefficient on the interaction between *female* and *CovInd* is remarkably similar to the one in our main specification in Table 7. This suggests that differences in sector and occupation distribution cannot explain the STW gender gap during the pandemic. In other words, within sector and occupation, female workers were still more likely to be put on STW than their male counterparts during the pandemic.^17^

Column (2) explores the role of the presence of children in the household. We include children dummies and their interactions with the female dummy and the COVID-19 index. We find that for men, the presence of children in the household does not significantly alter the probability of being on STW during COVID-19. For women with children, the effect on STW is negative. These results suggest that while women have been overall more likely to use STW during the COVID-19 crisis, the presence of children does not contribute to it. This result also confirms our finding in Table 4 that the presence of children did not amplify gender gaps during COVID-19.

Column (3) considers the role of telework availability of occupation on recourse to STW. We add the dummy variable *LowTele*, as defined in Sect. 5.6. The results show that having a job with low telework availability increases the probability of engaging in STW during the COVID-19 pandemic. However, this effect is significantly larger for women than for men, as can be seen in the positive and significant coefficient on the triple interaction term $female \times LowTele \times CovInd$. The STW gender gap is also significant in the high-teleworkable occupations but considerably smaller.

**Fig. 8:**
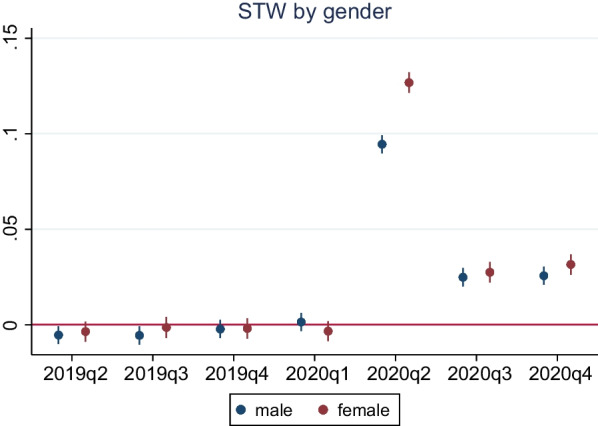
STW over time for men and women. *Notes* Estimates from regression (3) of STW dummy on quarterly dummies, run separately for men (blue) and women (red). Regressions estimated with linear probability model, including random effects

We further analyze how the probability of engaging in STW evolves through time by running regression (3) separately for men and women. The coefficient estimates of the quarterly dummies are plotted in Fig. 8. The figure shows that the effect of COVID-19 on the probability of being on STW is highest in the second quarter of 2020; it falls substantially in the third and fourth quarters but remains well above the pre-COVID levels. The figure also shows an economically large and statistically significant difference between men and women in the second quarter of 2020: Women’s probability of being in STW was about 3% higher than men’s in this quarter. The gender gap persists to some extent in the third and fourth quarters but is not statistically significant.

To sum up, the STW gender gap found after controlling for low telework availability suggests that women have disproportionately made use of STW. We do not know if this was the result of a request by female workers or the choice by the employer; nevertheless, it implies a disproportionate impact on female income since STW leads to a 20% reduction in salary.

## COVID-19 and hours worked

In this section, we only consider respondents who are currently employed or apprentices. We examine the effect of the COVID-19 crisis on (i) the probability of having effectively worked in the previous week and (ii) the number of hours worked in the previous week.

**Table 8:** The effect of COVID-19 and gender on having worked last week

	Worked last week			
	(1)	(2)	(3)	(4)
Female	−0.0286$$^{***}$$	−0.0250$$^{***}$$	−0.0180$$^{***}$$	−0.0346$$^{***}$$
	(0.00244)	(0.00442)	(0.00297)	(0.00280)
CovInd	−0.00903$$^{**}$$	−0.0287$$^{***}$$	−0.0187$$^{***}$$	0.0117$$^{**}$$
	(0.00440)	(0.0108)	(0.00539)	(0.00533)
Female $$\times$$ CovInd	−0.0269$$^{***}$$	−0.00609	−0.0223$$^{***}$$	−0.00983
	(0.00626)	(0.0122)	(0.00766)	(0.00748)
FullTime		0.0213$$^{***}$$		
		(0.00431)		
FullTime $\times$ female		0.0103$^{*}$		
		(0.00550)		
FullTime $$\times$$ index		0.0238$$^{**}$$		
		(0.0119)		
FullTime $\times$ female $\times$CovInd		−0.0279$^{*}$		
		(0.0154)		
Child0-6			−0.00870$^{*}$	
			(0.00462)	
Child7-14			−0.0171$$^{***}$$	
			(0.00480)	
Child0-6 $$\times$$ female			−0.0633$$^{***}$$	
			(0.00652)	
Child7-14 $\times$ female			−0.0104	
			(0.00661)	
Child0-6 $\times$ CovInd			0.0181	
			(0.0124)	
Child7-14 $$\times$$ CovInd			0.0460$$^{***}$$	
			(0.0127)	
Child0-6$\times$ female $\times$ CovInd			−0.00401	
			(0.0178)	
Child7-14$\times$ female $\times$ CovInd			−0.0189	
			(0.0180)	
LowTele				−0.0127$$^{***}$$
				(0.00364)
LowTele $$\times$$ female				0.0219$$^{***}$$
				(0.00500)
LowTele $$\times$$ CovInd				−0.0646$$^{***}$$
				(0.00943)
LowTele $$\times$$ female $$\times$$ CovInd				−0.0702$$^{***}$$
				(0.0137)
Constant	0.832$$^{***}$$	0.788$$^{***}$$	0.841$$^{***}$$	0.833$$^{***}$$
	(0.0381)	(0.0386)	(0.0402)	(0.00725)
Age FE	YES	YES	YES	YES
Canton FE	YES	YES	YES	YES
Education FE	YES	YES	YES	YES
NOGA FE	YES	YES	YES	YES
ISCO FE	YES	YES	YES	
Observations	151565	144373	142149	151478
$R^2$	0.0119	0.0143	0.0153	0.0131

Estimates from regression (2) of work last week on a constant, female dummy, COVID-19 stringency index, full-time dummy (column 2), child dummies (column 3), and low telework dummy (column 4). The sample is restricted to respondents who are employed or apprentices. Regressions estimated with linear probability model, including random effects. Standard errors in parentheses. Some insignificant estimates are eliminated for brevity

*p<0.1, **p<0.05, ***p<0.01

In the regressions presented in Table 8, the dependent variable is a dummy equal to 1 if the respondent did at least one hour of paid work in the previous week and 0 otherwise. Some employed respondents worked zero hours during the last week, possibly because they were on paid or unpaid leave, on STW, or working on an irregular schedule. The explanatory variables are the female dummy, the COVID-19 index, and their interaction. We control for age, education, occupation type, sector of work, and canton of residence. The results in column (1) show that, in normal times, women are less likely than men to have worked in the past week. During COVID-19, the probability of having worked in the past week fell for both men and women, but the effect is four times larger for women. This result is in line with the evidence that women are more likely to be in STW than men during COVID, documented in Sect. 6.2.

Column (2) introduces a full-time dummy that equals one if the respondent works full time and zero otherwise. The estimation results in Column (2) reveal that the probability of having worked in the past week fell similarly for both part-time working men and women during COVID-19. However, among full-time working respondents, women were more likely not to have worked than men during the pandemic. We speculate that part-time working women had sufficient flexibility to reconcile the additional family care needs imposed by the pandemic with their work schedule, while full-time working women lacked that flexibility and did not work.

Column (3) controls for the presence of children in the household. We find that, in normal times, the presence of children reduces the likelihood of having worked in the past week. The effect is strongest for women with children under 7 years old, suggesting that in normal times, a woman is more likely to take a leave of absence from work to take care of a child when it is needed. There is, however, no differential effect between men and women with children during COVID-19. Again, we speculate that men and women shared the additional childcare needs due to COVID-19 restrictions equally, or that women with children and part-time jobs had the flexibility to hold on to work, as documented in Sect. 3.

In the last column of Table 8, we consider the role of telework availability. In normal times, the gender gap in work probability is smaller in low-teleworkable occupations relative to high-teleworkable ones. During the pandemic, low teleworkability had a strong negative impact on the probability of having worked in the previous week for all respondents, and this effect is twice as large for women as for men. The effect could either come from the demand side, i.e., women in low-teleworkable occupations may have been disproportionately put on leave or STW by their employers, or from the supply side, i.e., women in those occupation may have asked to take leave during the pandemic.

**Table 9:** The effect of COVID-19 and gender on relative hours worked

	Hours worked last week over hours worked per contract			
	(1)	(2)	(3)	(4)
Female	0.00685	−0.00723	0.00611	−0.000381
	(0.00417)	(0.00718)	(0.00512)	(0.00477)
CovInd	−0.0726$$^{***}$$	−0.0883$$^{***}$$	−0.0778$$^{***}$$	−0.0678$$^{***}$$
	(0.00632)	(0.0154)	(0.00784)	(0.00755)
Female$\times$ CovInd	−0.0128	0.00923	−0.0115	−0.00209
	(0.00938)	(0.0177)	(0.0115)	(0.0111)
FullTime		−0.0467$$^{***}$$		
		(0.00677)		
FullTime $\times$ female		−0.00951		
		(0.00878)		
FullTime $\times$ CovInd		0.0184		
		(0.0169)		
FullTime $\times$ female$\times$ CovInd		−0.0377$^{*}$		
		(0.0222)		
Child0-6			−0.00992	
			(0.00763)	
Child7-14			−0.00897	
			(0.00791)	
Child0-6 $\times$ female			−0.00678	
			(0.0112)	
Child7-14 $\times$ female			0.000276	
			(0.0113)	
Child0-6 $\times$ CovInd			0.00784	
			(0.0177)	
Child7-14 $\times$ CovInd			0.0109	
			(0.0183)	
Child0-6 $\times$ female$\times$ CovInd			−0.0104	
			(0.0270)	
Child7-14 $\times$ female$\times$ CovInd			0.0128	

			(0.0271)	
LowTele				−0.00790
				(0.00605)
LowTele $$\times$$ female				0.0248$$^{***}$$
				(0.00849)
LowTele $\times$ CovInd				−0.0157
				(0.0139)
LowTele $$\times$$ female$$\times$$ CovInd				−0.0436$$^{**}$$
				(0.0210)
Constant	1.148$$^{***}$$	1.202$$^{***}$$	1.144$$^{***}$$	1.036$$^{***}$$
	(0.0809)	(0.0813)	(0.0851)	(0.0134)
Age FE	YES	YES	YES	YES
Canton FE	YES	YES	YES	YES
Education FE	YES	YES	YES	YES
NOGA FE	YES	YES	YES	YES
ISCO FE	YES	YES	YES	
Observations	116951	116724	109629	116636
$R^2$	0.00573	0.00754	0.00608	0.00496

Estimates from regression (2) of hours worked last week over per-contract hours worked on a constant, female dummy, COVID-19 stringency index, full-time dummy (column 2), child dummies (column 3), and low-teleworkable dummy (column 4). The sample is restricted to respondents who are employed or apprentices. Regressions estimated with linear model, including random effects. Standard errors in parentheses

*p<0.1, **p<0.05, ***p<0.01

We continue to test the effect of the pandemic on the absolute number of hours worked. The effect heavily depends on the occupation rate before the pandemic, as shown in Table 18 in Appendix. Therefore, we construct a relative hours worked variable and use it as the dependent variable in Table 9. The relative hours worked variable is defined as the number of hours worked in the previous week divided by per-contract hours worked.^18^ Column (1) indicates that in relative terms, there is no differential impact between male and female hours worked during the pandemic.

Column (2) introduces the full-time dummy and its interactions with the female and COVID index dummies. The estimation results reveal that full-time working women cut their relative hours worked more than men during the pandemic. There is, however, no similar gender gap for part-time workers. This result supports the argument that part-time working women had sufficient flexibility to reconcile the additional childcare needs with their work schedule and therefore were comparatively less affected. Full-time working women did not have the same flexibility, making it more challenging to continue working the usual hours during the pandemic.

Column (3) assesses how the presence of children affected relative hours worked during the pandemic. It indicates that children did not significantly affect relative hours worked during COVID, neither for men nor for women. Finally, column (4) considers the role of teleworkability on hours worked during the pandemic; it shows that women in low-teleworkable occupations experienced a more significant decrease in hours worked than their male counterparts. The pandemic had no differential effect on men and women in high-teleworkable occupations.

## The gender wage gap

**Fig. 9:**
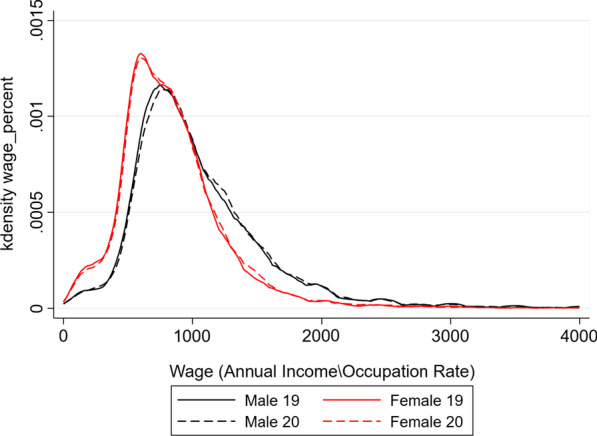
Wage distribution by gender and year. *Notes* This figure plots kernel density of the wage distribution of men and women in 2019 and 2020. Wage is measured by dividing income by the occupation rate. The black line is for men and red line is for women. The solid and dashed lines correspond to the data in 2019 and 2020, respectively

We finally ask if the COVID-19 pandemic affected wages of men and women differently. We measure wage by dividing a respondent’s annual income by his or her occupation rate.^19^ Figure 9 shows the wage distribution of male and female respondents in 2019 and 2020. In both years, men’s wage distribution is situated to the right of women’s, indicating that men earn on average more than women after controlling for the occupation rate. In addition to this, men’s distribution is more heavily skewed to the right, indicating a bigger fraction of men among the high earners. Similarly, the wage distribution indicates a higher fraction of women among low earners. The figure also shows that, for low wage-occupancy rate levels, the male distribution shifted (somewhat) rightward in 2020, while the female distribution did not; for higher wage-occupancy rate levels, both male and female distributions shifted rightward.

**Fig. 10:**
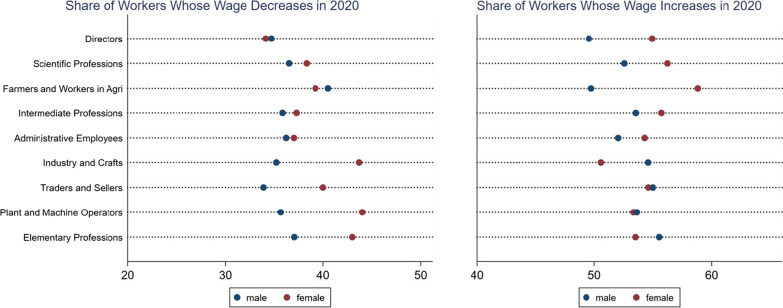
Wage changes by gender and occupation. *Notes* We restrict the sample to respondents with wage information (annual income/occupation rate, unit: CHF per percentage occupation rate) in 2019 and 2020, calculate the percentage of respondents whose wage has decreased/increased from 2019 to 2020, and then break it down by occupation types and gender. Occupations are ranked in descending order of telework availability in 2020

Figure 10 documents how wages evolved during the COVID-19 pandemic across gender and occupations. We restrict the sample to respondents with wage information available in both 2019 and 2020 and plot the percentage of workers who experienced wage decrease (left panel) and increase (right panel) between 2019 and 2020 by gender and occupation. In Fig. 10, occupations are ranked in descending teleworkability order. The figure indicates that, in low-teleworkable occupations, women were more likely than men to experience a wage reduction during the COVID-19 period, indicating an increase in the gender wage gap. This decline in wages may relate to the recourse to STW during the pandemic: Employees in STW maintain their employment contract, and therefore also their contractual occupation rate, but receive compensation of only 80% for the reduced working hours. The wage gap result is thus consistent with our previous insights on STW, namely that women, particularly in low-teleworkable occupations, were more likely to be put on STW than their male counterparts and, therefore, suffer a wage drop. Interestingly, in high-teleworkable occupations, women were more likely than men to experience a wage increase during COVID-19. These opposite effects on women’s wages are consistent with the evidence on women’s wage distribution in 2020 reported in Fig. 9.

## Conclusion

The COVID-19 crisis generated an unprecedented decline in economic activity, employment and hours worked. This paper studies the effect of COVID-19 on labor market gender gaps in Switzerland. In particular, we find that women were more likely to exit the labor force during the peak of the pandemic than their male counterparts. An important explanation for this effect is that women were overly present in sectors and occupations that were hit particularly hard. This result may have long-lasting consequences if non-active women see their skills depreciate, find it harder to regain employment or must accept lower wages after the crisis.

Our results also point to a significant role played by telework feasibility. We reveal significant heterogeneity in the effects of the pandemic on labor market outcomes depending on the availability of telework. Gender gaps in labor market participation, STW, and hours worked were significantly larger in occupations where telework was not feasible. Women in low-teleworkable occupation experienced wage declines more often than their male counterparts, while women in high-teleworkable occupation more often benefited from an increase in their wage. This result has significant implications also for the future. We can conjecture that the heterogeneity in the gender gaps will persist and that women in high-teleworkable occupations will face more favorable conditions in the future, with increased opportunities to work from home, higher flexibility, and easier reconciliation of career and family life. On the other hand, women in low-teleworkable occupations are left more vulnerable after the pandemic.

In addition, our study show specificities of the Swiss labor market that mitigated the negative effect of the crisis on women. Contrary to the USA, there was no gender gap in unemployment during the pandemic in Switzerland, but we find a significant gender gap in the recourse to STW. The Swiss government greatly facilitated the use of STW during the pandemic; this policy likely prevented many layoffs, and in particular for women, but also reduced their wage.

Marriage and the presence of children in the household did not amplify the labor market participation gender gap during the crisis. This result contrasts with findings in the USA, where mothers were disproportionately affected. This result can be explained by several factor. First, the widespread use of STW policy allowed women to maintain their employment while reducing hours worked to care for children. Second, school closure in Switzerland was much shorter than in the USA and many other countries. Finally, the high percentage of women working part-time points to more flexibility for women in the Swiss labor market, allowing them to continue working during the pandemic.

## Figures and Tables

**Fig. 11:**
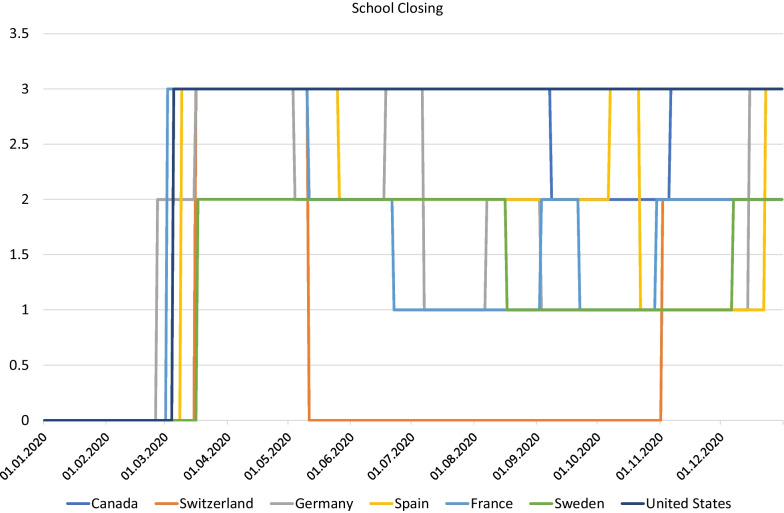
School closure index. *Notes* The index records the stringency of school closure policy in several developed economies. The values range from 0 (=no measures) to 3 (= closing all levels). *Source* Oxford

## Data Availability

The SLFS data that support the findings of this study are available from the Swiss Federal Statistical Office, but restrictions apply to the availability of these data, which were used under license for the current study, and so are not publicly available. Data are, however, available from the authors upon reasonable request and with permission of Federal Statistical Office.

## Appendix

### Definition of variables

*KOF stringency index*: The indices are composite measures including different lockdown policies, such as school and workplace closure. The values range from 0 (= no measures) to 100 (= full lockdown). The data are available at the national level and for all individual 26 cantons of Switzerland from January 2020 onward. The construction largely follows the code book of the Oxford COVID-19 Government Response Tracker.^20^

*Gender* (BB04A): male, female.

*Labor market status* (B0000): active, apprentice, unemployed according to the ILO, non-active.

*Age category* (AGE64): 15–24, 25–39, 40–54, 55–64, 65+.

*Education* (TBQ2): Highest education level achieved: middle school, high school or equivalent, college.

*Canton* (B017): canton of residence.

*Occupation* (BFU7): Occupation according to the International Standard Classification of Occupations (ISCO-08 at 1 position). The variable refers to current occupation for employed respondents and to previous occupation for unemployed and inactive ones. Respondents who were never active or have been inactive for more than 8 years do not answer this question.

*Sector* (BMU3): Sector according to the General Classification of Economic Activities classification (NOGA-08 level 1). The variable refers to current sector for employed respondents and to previous sector for unemployed and inactive ones. Respondents who were never active or have been inactive for more than 8 years do not answer this question.

*Civil status* (IS03): single, married, divorced, widower, in a registered partnership, separated, other.

*Children* (FAMTYP2): The variable refers to the presence of children in the household, either own or partner’s child. No child under 15, youngest child aged 7–14, youngest child aged 0-6.

*Work from home* (EI04): Binary variable recording whether the respondent worked from home at least once over the last four weeks.

*Reason for reduction in hours of work* (EK101): Respondents who experienced a reduction in hours worked in the previous week provide a reason for the reduction (vacation, military service, maternity/paternity leave, sick leave, education, family responsibilities, STW, personal, weather, variable hours, compensation of overtime, other). The variable is used to construct the STW dummy and the family leave dummy.

*Job search* (BD08): Respondents that are not currently employed are asked whether they were searching for a job in the last 4 weeks.

*Reasons for not searching job* (BD131, BD132, BD133): Respondents that are not currently employed and not currently searching for a job are asked to state the reasons why they are not searching. Education, military service, retirement, sickness, invalidity, child care, other personal or family responsibilities, other.

*Worked last week* (BD01): The variable records whether the respondent performed at least one hour of paid work in the previous week.

*Hours worked last week* (EK08): Number of hours effectively worked in the previous week.

*Occupation rate in percent* (EK08)

*Annual income* (BWU1): Gross annual professional income.

### School closure policies

The Oxford COVID-19 Government Response Tracker (OxCGRT) collects information on school closure policies across countries and summarizes them on a scale from 0 to 3 reflecting the stringency of the measures. In the data, 0: no measures; 1: recommend closing or all school open with alterations resulting in significant differences compared to non-COVID-19 operations; 2: require closing (only some levels or categories, e.g., just high school or public schools); and 3: require closing all levels.

Figure 11 shows that Switzerland has a more lenient school closure policy. Swiss schools reopened in May 2020, while other countries kept strict measures throughout 2020.

### Additional tests

#### Robustness

Table 10 presents a robustness check where we only include respondents for which we have complete information on occupation type (ISCO) and economic sector (NOGA). For non-active respondents, this question is only answered by participants who had been employed over the previous 8 years. The sample in this robustness check therefore excludes non-active respondents who never worked or exited the labor market more than 8 years ago. The regression estimates are consistent with those in the main text (Table 1).

**Table 10:** Robustness: COVID-19 and labor market status

	Employed	Unemployed	Non-active
Female	−0.0402$$^{***}$$	0.00104	0.0375$$^{***}$$
	(0.00254)	(0.00142)	(0.00218)
CovInd	−0.0148$$^{***}$$	0.00650$$^{***}$$	0.00928$$^{**}$$
	(0.00288)	(0.00204)	(0.00244)
Female $$\times$$ CovInd	−0.00502	−0.00310	0.00805$$^{**}$$
	(0.00406)	(0.00287)	(0.00343)
Constant	0.713$$^{***}$$	0.0324	0.253$$^{***}$$
	(0.0421)	(0.0230)	(0.0362)
Age FE	YES	YES	YES
Canton FE	YES	YES	YES
Education FE	YES	YES	YES
NOGA FE	YES	YES	YES
ISCO FE	YES	YES	YES
Observations	170619	170619	170619
$R^2$	0.0422	0.0108	0.0445

Estimates from regression (1) of labor market status on a constant, female dummy, COVID-19 stringency index, and its interaction with the female dummy. The sample is restricted to respondents for which we have complete information on occupation type (ISCO) and economic sector (NOGA). Regressions estimated with linear probability model, including random effects. Robust standard errors in parentheses

**p*<0.1, ***p*<0.05, ****p*<0.01

Table 11 presents a robustness check where we consider individual fixed effects. The results are overall robust. By running this type of regression, we focus on the variation over time and cannot include the variables that do not vary over time, like female.

**Table 11:** Robustness: individual fixed effects

	Employed	Unemployed	Non-active	In STW	Worked last week	Hours worked
CovInd	−0.0179$$^{***}$$	0.00186	0.0160$$^{***}$$	0.109$$^{***}$$	−0.0121$$^{**}$$	−2.925$$^{***}$$
	(0.00305)	(0.00251)	(0.00279)	(0.00287)	(0.00555)	(0.260)
Female $$\times$$ CovInd	−0.00399	−0.00349	0.00748$$^{*}$$	0.0177$$^{***}$$	−0.0262$$^{***}$$	0.259
	(0.00419)	(0.00344)	(0.00382)	(0.00394)	(0.00762)	(0.356)
Constant	0.636$$^{***}$$	−0.0238	0.388$$^{***}$$	0.0279	0.547$$^{***}$$	10.46
	(0.0953)	(0.0782)	(0.0869)	(0.0897)	(0.173)	(8.113)
Age FE	YES	YES	YES	YES	YES	YES
Canton FE	YES	YES	YES	YES	YES	YES
Education FE	YES	YES	YES	YES	YES	YES
NOGA FE	YES	YES	YES	YES	YES	YES
ISCO FE	YES	YES	YES	YES	YES	YES
Individual FE	YES	YES	YES	YES	YES	YES
Observations	186881	186881	186881	186881	186861	186881
$R^2$	0.352	0.0125	0.407	0.0117	0.187	0.133

Estimates from regression (1) of labor market status, being in STW, worked last week and hours on a constant, COVID-19 index, and its interaction with the female dummy. Regressions estimated with linear probability model, including individual fixed effects. Robust standard errors in parentheses

**p*<0.1, ***p*<0.05, ****p*<0.01

As an additional robustness, we run regression (1) replacing the COVID-19 index by a COVID-19 dummy that equals zero from 2019Q1 to 2020Q1 and one from 2020Q2 to 2020Q4. Table 12 shows the estimates when taking labor market statuses, being in STW, worked last week and hours as dependent variables, and Table 13 reports the results when taking non-active as dependent variables, and considering the effect of being married, having children and working with low teleworkability. The results are broadly consistent with those in the main text. Finally, Table 14 displays estimates from regression (1) taking STW, work last week, hours worked, and family leave as dependent variables and including $NOGA \times CovInd$ and $ISCO\times CovInd$ fixed effects.

**Table 12:** Robustness: replacing COVID stringency index by COVID dummy

	Employed	Unemployed	Non-active	In STW	Worked last week	Hours worked
Female	−0.0399$$^{***}$$	−0.00247$$^{*}$$	0.0412$$^{***}$$	0.00218	−0.0292$$^{***}$$	−8.991$$^{***}$$
	(0.00230)	(0.00137)	(0.00203)	(0.00135)	(0.00238)	(0.126)
Cov	−0.00763$$^{***}$$	0.00348$$^{***}$$	0.00470$$^{***}$$	0.0517$$^{***}$$	−0.0128$$^{***}$$	−1.466$$^{***}$$
	(0.00151)	(0.00114)	(0.00137)	(0.00133)	(0.00230)	(0.101)
Female $$\times$$ Cov	−0.00328	−0.000605	0.00336$$^{*}$$	0.0137$$^{***}$$	−0.0176$$^{***}$$	0.270$$^{*}$$
	(0.00210)	(0.00158)	(0.00190)	(0.00191)	(0.00329)	(0.146)
Constant	0.726$$^{***}$$	0.0291	0.244$$^{***}$$	0.0188	0.788$$^{***}$$	50.15$$^{***}$$
	(0.0396)	(0.0233)	(0.0350)	(0.0220)	(0.0390)	(2.117)
Age FE	YES	YES	YES	YES	YES	YES
Canton FE	YES	YES	YES	YES	YES	YES
Education FE	YES	YES	YES	YES	YES	YES
NOGA FE	YES	YES	YES	YES	YES	YES
ISCO FE	YES	YES	YES	YES	YES	YES
Observations	186881	186881	186881	158250	158242	133583
$R^2$	0.417	0.0423	0.459	0.0392	0.0201	0.223

Estimates from regression (1) of labor market status, being in STW, worked last week and hours on a constant, female dummy, COVID-19 dummy, and its interaction with the female dummy. Regressions estimated with linear probability model, including random effects. Robust standard errors in parentheses

**p*<0.1, ***p*<0.05, ****p*<0.01

**Table 13:** Robustness: replacing COVID stringency index by COVID dummy

	Non-active dummy		
Female	0.00773$$^{**}$$	0.0198$$^{***}$$	0.0366$$^{***}$$
	(0.00307)	(0.00260)	(0.00246)
Cov	0.000666	0.00429$$^{***}$$	0.00327$$^{**}$$
	(0.00204)	(0.00161)	(0.00162)
Female $$\times$$ Cov	0.00989$$^{***}$$	0.00996$$^{***}$$	0.00251
	(0.00285)	(0.00227)	(0.00225)
Married	−0.0180$$^{***}$$		
	(0.00294)		
Married $$\times$$ female	0.0548$$^{***}$$		
	(0.00392)		
Married $$\times$$ Cov	0.00588$$^{**}$$		
	(0.00269)		
Married $$\times$$ female $$\times$$ Cov	−0.00915$$^{**}$$		
	(0.00378)		
Child0-6		−0.0179$$^{***}$$	
		(0.00399)	
Child7-14		−0.0196$$^{***}$$	
		(0.00406)	
Child0-6 $$\times$$ female		0.0866$$^{***}$$	
		(0.00547)	
Child7-14 $$\times$$ female		0.0263$$^{***}$$	
		(0.00556)	
Child0-6 $\times$ Cov		−0.000867	
		(0.00382)	
Child7-14 $\times$ Cov		−0.00131	
		(0.00391)	
Child0-6 $$\times$$ female $$\times$$ Cov		−0.0193$$^{***}$$	
		(0.00532)	
Child7-14 $$\times$$ female $$\times$$ Cov		−0.0126$$^{**}$$	
		(0.00548)	
LowTele			0.00534$^{*}$
			(0.00304)
LowTele $$\times$$ female			0.0198$$^{***}$$
			(0.00407)
LowTele $\times$ Cov			0.00252
			(0.00286)
LowTele $$\times$$ female $$\times$$ Cov			0.00825$$^{**}$$
			(0.00409)
Constant	0.269$$^{***}$$	0.259$$^{***}$$	0.0943$$^{***}$$
	(0.0362)	(0.0376)	(0.00676)
Observations	170619	160033	170516
$R^2$	0.0472	0.0494	0.0429

Estimates from regression (2) of non-active dummy on a constant, female and COVID-19 dummies, and their interaction with marriage dummy (column1), children dummies (column 2) and low teleworkability dummy (column 3). Regressions estimated with linear probability model, including random effects. Standard errors in parentheses

**p*<0.1, ***p*<0.05, ****p*<0.01

**Table 14:** Robustness: adding NOGA $\times$ CovInd and ISCO $\times$ CovInd

	in STW	Worked last week	Hours worked	Family leave
Female	0.000907	−0.0321$$^{***}$$	−8.934$$^{***}$$	0.00210$$^{***}$$
	(0.00144)	(0.00255)	(0.131)	(0.000442)
CovInd	0.125	0.0212	−2.248	0.000953
	(0.0864)	(0.150)	(6.298)	(0.0275)
Female $$\times$$ CovInd	0.0286$$^{***}$$	−0.0177$$^{**}$$	0.223	0.00275$$^{**}$$
	(0.00401)	(0.00695)	(0.303)	(0.00129)
Constant	0.0188	0.788$$^{***}$$	50.12$$^{***}$$	0.000762
	(0.0220)	(0.0390)	(2.449)	(0.00652)
Age FE	YES	YES	YES	YES
Canton FE	YES	YES	YES	YES
Education FE	YES	YES	YES	YES
NOGA FE	YES	YES	YES	YES
ISCO FE	YES	YES	YES	YES
NOGA $\times$ CovInd FE	YES	YES	YES	YES
ISCO $\times$ CovInd FE	YES	YES	YES	YES
Observations	158250	158242	133583	158250
$R^2$	0.0577	0.0227	0.224	0.00224

Estimates from regression (1) of STW (column 1), work last week (column 2), relative hours worked last week (column 3) and family leave (column 4) on a constant, female dummy and COVID-19 stringency index. The sample is restricted to respondents who are employed or apprentices. Regressions estimated with linear probability model, including random effects. Standard errors in parentheses

**p*<0.1, ***p*<0.05, ****p*<0.01

#### COVID-19 and individual telework availability

First, we report evidence that education and telework availability are positively correlated. In the survey we use, employed respondents are asked if they have worked remotely in the last four weeks. We calculate the fraction of respondents, by educational level, who regularly, occasionally, or never work remotely, which is reported in Table 15.

Second, we report the fraction of respondents, by gender, who regularly, occasionally, or never work remotely, in Table 16. Men have a higher chance of working from home than women. We further tested the difference by running a regression of individual teleworkability on gender and found that it is significant.

To assess the effect of telework on gender inequalities much more thoroughly, we redo the test in Sect. 5.6 by replacing the occupational teleworkable dummy with an individual teleworkable dummy. The estimated results are presented in Table 17. At the individual level, we still find that among those who cannot work from home, women were less likely to be employed and more likely to become non-active than men during COVID. After checking the significance of the composite coefficient (”female $\times$ CovInd” and ”Remote $\times$ female $\times$ CovInd”), women and men turn out to be not differently affected by the pandemic if they can work from home. This result confirms our previous finding in Sect.  5.6 that the non-active gender gap has mainly widened for respondents who has low teleworkability.

**Table 15:** Telework availability and educational level

	Remote working			
Education	No	Occasionally	Regularly	Total
Middle school	92.53	3.94	3.53	100
High school	76.06	13.29	10.62	100
College	44.86	29.03	26.11	100
Total	62.97	19.8	17.23	100

**Table 16:** Telework availability by gender

	Remote working			
Gender	No	Occasionally	Regularly	Total
Male	60.11	22.83	17.06	100
Female	65.86	16.99	17.15	100

Results are reported in percentage points

**Table 17:** COVID-19, labor market status, and individual telework availability

	Employed	Unemployed	Non-active
Female	−0.00297$$^{**}$$	0.000690	0.00228$$^{**}$$
	(0.00128)	(0.000831)	(0.00102)
CovInd	−0.0352$$^{***}$$	0.0140$$^{***}$$	0.0212$$^{***}$$
	(0.00262)	(0.00171)	(0.00209)
Female $$\times$$ CovInd	−0.00757$$^{**}$$	−0.00260	0.0102$$^{***}$$
	(0.00365)	(0.00238)	(0.00292)
Remote	0.00472$$^{***}$$	−0.00291$$^{***}$$	−0.00181
	(0.00152)	(0.000989)	(0.00121)
Remote $\times$ female	0.00141	0.000791	−0.00220
	(0.00216)	(0.00141)	(0.00172)
Remote $$\times$$ CovInd	0.0216$$^{***}$$	−0.00501$$^{*}$$	−0.0166$$^{***}$$
	(0.00420)	(0.00274)	(0.00335)
Remote $\times$ female $\times$ CovInd	0.00195	0.00322	−0.00517
	(0.00611)	(0.00398)	(0.00488)
Constant	0.944$$^{***}$$	0.00796$$^{***}$$	0.0485$$^{***}$$
	(0.00353)	(0.00230)	(0.00282)
Age FE	YES	YES	YES
Canton FE	YES	YES	YES
Education FE	YES	YES	YES
NOGA FE	YES	YES	YES
Observations	133926	133926	133926
$R^2$	0.164	0.0135	0.182

We show estimates from regression (2) of labor market status on a constant, female dummy (1 for women and 0 otherwise), COVID-19 stringency index, remote dummy (1 for respondents who can perform remote working occasionally or regularly and 0 otherwise), and their interactions. Regressions estimated with linear probability model, including random effects. Robust standard errors in parentheses

**p*<0.1, ***p*<0.05, ****p*<0.01

#### COVID-19 and hours worked

In Table 18, we analyze how the COVID-19 pandemic affected the respondents’ hours worked. The dependent variable is the respondent’s effective hours worked in the past week. The explanatory variables are the female dummy, the COVID-19 index, and their interaction. We control for age, education, occupation type, sector of work, and canton of residence. Column (1) shows that women worked about 9 hours per week less than men in normal times; during the pandemic, all respondents reduced their hours worked, but women reduced them less than men. This result may simply be driven by the fact that women typically hold a part-time job and cannot reduce hours as much as men.

Column (2) estimates how the presence of children in the household affects hours worked during normal and pandemic times. The results show that in normal times, the presence of children, whether pre-school or school-aged, significantly impacts mothers’ working hours, while it barely affects that of fathers. Women without children work about 6.6 hours less than their male counterparts on average, but the gender gap more than doubles for mothers of children under 15. During the pandemic, the evidence suggests that the presence of school-aged children did not significantly impact hours worked by fathers and mothers. We find that mothers of younger children experienced a lower decrease in hours worked, most likely because they were working fewer hours before the pandemic.

**Table 18:** The effect of COVID-19 and gender on hours worked

	Hours worked last week	
	(1)	(2)
Female	−8.999$$^{***}$$	−6.626$$^{***}$$
	(0.128)	(0.152)
CovInd	−2.979$$^{***}$$	−3.067$$^{***}$$
	(0.187)	(0.229)
Female $\times$ CovInd	0.514$^{*}$	0.120
	(0.270)	(0.330)
Child0-6		−0.585$$^{**}$$
		(0.232)
Child7-14		0.426$^{*}$
		(0.240)
Child0-6 $$\times$$ female		−8.887$$^{***}$$
		(0.332)
Child7-14 $$\times$$ female		−7.430$$^{***}$$
		(0.333)
Child0-6 $\times$ CovInd		−0.0668
		(0.522)
Child7-14 $\times$ CovInd		0.552
		(0.537)
Child0-6 $\times$ female $\times$ CovInd		1.417$^{*}$
		(0.771)
Child7-14 $\times$ female $\times$ CovInd		1.015
		(0.770)
Constant	50.21$$^{***}$$	51.05$$^{***}$$
	(2.117)	(2.156)
Age FE	YES	YES
Canton FE	YES	YES
Education FE	YES	YES
NOGA FE	YES	YES
ISCO FE	YES	YES
Observations	133583	125218
$R^2$	0.223	0.250

Estimates from regression (2) of hours worked last week on a constant, female dummy, COVID-19 stringency index, and child dummies (column 2). The sample is restricted to respondents who are employed or apprentices. Regressions estimated with linear model, including random effects. Standard errors in parentheses

**p*<0.1, ***p*<0.05, ****p*<0.01

#### COVID-19 and family leave

The Swiss federal government imposed complete school closure between March 16 and May 11, 2020 (8 weeks). It is possible that some workers would request a family leave to care for their children or elderly parents. Table 19 displays estimates from a regression whose dependent variable is a dummy with value one if the person was on family leave in the previous week and zero otherwise. Employed respondents are said to be on family leave if they did not work last week and state that this absence is due to family responsibilities. Family leave can refer to a paid or unpaid leave.

**Table 19:** The effect of COVID-19 and gender on family leave

	Request a family leave		
	(1)	(2)	(3)
Female	0.00236$$^{***}$$	0.00138$$^{***}$$	0.00268$$^{***}$$
	(0.000422)	(0.000518)	(0.000488)
CovInd	0.00238$$^{***}$$	−0.000105	0.00306$$^{***}$$
	(0.000802)	(0.000973)	(0.000972)
Female $\times$ CovInd	0.00157	0.000102	0.00172
	(0.00115)	(0.00139)	(0.00137)
Child0-6		0.00275$$^{***}$$	
		(0.000827)	
Child7-14		0.00227$$^{***}$$	
		(0.000858)	
Child0-6 $$\times$$ female		0.00667$$^{***}$$	
		(0.00117)	
Child7-14 $\times$ female		0.00148	
		(0.00118)	
Child0-6 $$\times$$ CovInd		0.0151$$^{***}$$	
		(0.00230)	
Child7-14 $\times$ CovInd		0.00247	
		(0.00237)	
Child0-6 $\times$ female $\times$ CovInd		0.000233	
		(0.00331)	
Child7-14 $$\times$$ female $$\times$$ CovInd		0.00800$$^{**}$$	
		(0.00335)	
LowTele			0.000956
			(0.000631)
LowTele $\times$ female			−0.00143
			(0.000869)
LowTele $\times$ CovInd			−0.00213
			(0.00172)
LowTele $\times$ female $\times$ CovInd			−0.000850
			(0.00251)
Constant	0.000685	0.00260	0.00190
	(0.00652)	(0.00701)	(0.00118)
Age FE	YES	YES	YES
Canton FE	YES	YES	YES
Education FE	YES	YES	YES
NOGA FE	YES	YES	YES
ISCO FE	YES	YES	
Observations	158250	148431	158162
$R^2$	0.00191	0.00600	0.00193

Estimates from regression (2) of family leave dummy on a constant, female dummy, COVID-19 stringency index, children dummies (column 2), and low telework dummy (column 3). The sample is restricted to respondents who are employed or apprentices. Regressions estimated with linear probability model, including random effects. Standard errors in parentheses

**p*<0.1, ***p*<0.05, ****p*<0.01

Column (1) displays regression results, including the COVID-19 index, the female dummy, and their interaction, as well as the usual labor market controls. The estimates emphasize that, in normal times, women are more likely to be on family leave. The COVID-19 crisis significantly increased the recourse to family leave for both men and women, but without any significant difference between the two.

Column (2) addresses the role of children in the household. We include children dummies and their interactions with the female dummy and the COVID-19 index. During the COVID-19 crisis, having children under 7 increased the probability of being on family leave without any significant difference between men and women. However, in households with children aged 7 to 14, women were more likely to be on family leave during the crisis, possibly reflecting the effect of school closures. Column (3) considers teleworkability and indicates that the latter affects family leave neither in normal nor in COVID-19 times.

## Abbreviations

STW: Short-time work
SLFS: The Swiss Labor Force Survey
SECO: The State Secretariat for Economic Affairs
NOGA: The General Classification of Economic Activities
ISCO: The International Standard Classification of Occupations

## Footnotes

1 Unlike other countries in Europe, such as Italy and Greece, the Swiss government did not introduce any temporary policy to prevent firms to fire workers during the COVID lockdown. However, it expanded and simplified its STW policy.

2 The interviews are conducted with a gap of 3 months between the first and the second interview, 9 months between the second and the third, and 3 months again between the third and the fourth.

3 See Appendix for more details.

4 The correlation between the stringency index of any two Swiss cantons is above 0.95, showing a high degree of similarity of COVID-19 policies across cantons. On this basis, we use the national stringency index.

5 In Sect. 9 of Appendix, we replace the KOF stringency index with a COVID dummy equal to one for quarters two, three, and four of 2020 and zero in quarter 1 of 2020 and report the average treatment effect of the pandemic on the gender gaps in labor market outcomes.

6 We assume that the lockdown stringency affects labor market outcomes contemporaneously, as closures of schools, shops, and places of work instantaneously impact the capacity and the incentive to work.

7 About half of the non-active respondents do not report previous occupation and sector; they are nonetheless included in all the regressions in this section. Our results are robust to only including participants with information on sector and occupation, as shown in Sect. 9.

8 As the COVID stringency index went up from 0 in the pre-COVID-19 periods to 0.75 at the peak of the pandemic, women were 0.43 percentage point (0.00583*0.75) more likely to be non-active than men.

9 We consider transitions between different labor market statuses in Sect. 5.2.

10 We discuss the gendered effects of the STW policy in Sect. 6.2.

11 We calculate the probabilities in panel (b) by taking the average of quarter-to-quarter transition probabilities in 2020Q3 and 2020Q4, and then taking the difference relative to the average in 2019Q3 and 2019Q4.

12 The sum of the two coefficients, “female $\times$ CovInd” and “married $\times$ female $\times$ CovInd” measures the impact of the pandemic on the gender gap among married people. The test on the joint significance reveals that the sum is not significantly different from zero.

13 The children dummies only consider the age of the youngest child in the household.

14 We test the joint significance of the sum of the coefficients on “female $\times$ CovInd” and “child0-6 $\times$ female $\times$ CovInd” and find it is significantly different from zero; the sum of the coefficients on “female $\times$ CovInd” and “child7-14 $\times$ female $\times$ CovInd” is not significantly different from zero.

15 See Fig. 11 for the stringency of school closure policy; Switzerland reopened its schools in May 2020, while other countries kept strict measures throughout 2020.

16 In Sect. 9 of Appendix, we repeat this exercise by replacing the occupational teleworkable dummy with an individual teleworkable measure and find similar results.

17 Note that we find no evidence that the STW gender gap is related to the lower pre-pandemic occupation rate of women. Women are more likely to be put on STW than men, regardless of working full-time or part-time. Results are available upon request.

18 Per-contract hours worked are replaced by usual hours worked for independent employees.

19 Ideally, we would calculate wage per hour by dividing annual income by annual hours worked. Data on annual hours is not available, so we use the occupation rate as a proxy.

## References

[CR1] Abraham KG, Houseman SN (2014). Short-time compensation as a tool to mitigate job loss? Evidence on the us experience during the recent recession. Industrial Relations: A Journal of Economy and Society.

[CR2] Adams-Prassl A, Boneva T, Golin M, Rauh C (2020). Inequality in the impact of the coronavirus shock: Evidence from real time surveys. Journal of Public Economics.

[CR3] Albanesi S, Kim J (2021). Effects of the covid-19 recession on the us labor market: Occupation, family, and gender. Journal of Economic Perspectives.

[CR4] Alon, T. M., Doepke, M., Olmstead-Rumsey, J., & Tertilt, M. (2020). The impact of covid-19 on gender equality. In *Technical report* National Bureau of economic research.

[CR5] Alon T, Coskun S, Doepke M, Koll D, Tertilt M (2022). From mancession to shecession: Women’s employment in regular and pandemic recessions. NBER Macroeconomics Annual.

[CR6] Andrew, A., Cattan, S., Costa Dias, M., Farquharson, C., Kraftman, L., Krutikova, S., Phimister, A., Sevilla, A. (2020). The gendered division of paid and domestic work under lockdown. In *IZA Discussion* Paper No. 13500.

[CR7] Benzeval, M., Burton, J., Crossley, T.F., Fisher, P., Jäckle, A., Low, H., Read, B. (2020). *The idiosyncratic impact of an aggregate shock: The distributional consequences of covid-19*. Available at SSRN 3615691.

[CR8] Bick, A., Blandin, A. (2021). *Real-time labor market estimates during the 2020 coronavirus outbreak*. Available at SSRN 3692425

[CR9] Bluedorn, J., Caselli, F., Hansen, N.-J., Shibata, I., Tavares, M. (2021). Gender and employment in the covid-19 recession: Evidence on “she-cessions”. In *Technical report, IMF working paper*.

[CR10] Cajner, T., Crane, L. D., Decker, R. A., Grigsby, J., Hamins-Puertolas, A., Hurst, E., Kurz, C., & Yildirmaz, A. (2020). The us labor market during the beginning of the pandemic recession. In *Technical report*. National Bureau of Economic Research.

[CR11] Cajner, T., Crane, L.D., Decker, R., Hamins-Puertolas, A., Kurz, C.J. (2020a). Tracking labor market developments during the covid-19 pandemic: A preliminary assessment. In *FEDS working paper No. 2020-030*.

[CR12] Coibion, O., Gorodnichenko, Y., & Weber, M. (2020). *Labor markets during the covid-19 crisis: A preliminary view*. National Bureau of Economic Research: Technical report.

[CR13] Collins C, Landivar LC, Ruppanner L, Scarborough WJ (2021). Covid-19 and the gender gap in work hours. Gender, Work & Organization.

[CR14] Couch, K.A., Fairlie, R.W., Xu, H. (2020). *Gender and the COVID-19 Labor Market Downturn*. Stanford Institute for Economic Policy Research (SIEPR).

[CR15] Del Boca D, Oggero N, Profeta P, Rossi M (2020). Women’s and men’s work, housework and childcare, before and during covid-19. Review of Economics of the Household.

[CR16] Fabrizio, M.S., Gomes, D.B., Tavares, M.M.M. (2021). COVID-19 *She-Cession: The employment penalty of taking care of young children*. International Monetary Fund.

[CR17] Farré, L., Fawaz, Y., González, L., Graves, J. (2020) How the covid-19 lockdown affected gender inequality in paid and unpaid work in Spain. In *IZA Discussion* paper No. 13434.

[CR18] Forsythe E, Kahn LB, Lange F, Wiczer D (2020). Labor demand in the time of covid-19: Evidence from vacancy postings and ui claims. Journal of public economics.

[CR19] Gupta, S., Montenovo, L., Nguyen, T.D., Lozano-Rojas, F., Schmutte, I.M., Simon, K.I., Weinberg, B.A., Wing, C. (2020). Effects of social distancing policy on labor market outcomes. NBER Working paper (w27280). 10.1111/coep.12582 PMC10635474 37946719

[CR20] Hijzen, A., Venn, D. (2011). The role of short-time work schemes during the 2008-09 recession. OECD Social, Employment and Migration Working Papers, No. 115.

[CR21] Juranek S, Paetzold J, Winner H, Zoutman F (2021). Labor market effects of covid-19 in sweden and its neighbors: Evidence from administrative data. Kyklos.

[CR22] Kopp D, Siegenthaler M (2017). Does short-time work prevent unemployment?. Labor Market Policy.

[CR23] Leibovici, F., Santacreu, A. M., & Famiglietti, M. (2020). *Social distancing and contact-intensive occupations*. St. Louis FED: On the economy.

[CR24] Mongey S, Pilossoph L, Weinberg A (2021). Which workers bear the burden of social distancing?. The Journal of Economic Inequality.

[CR25] Montenovo, L., Jiang, X., Rojas, F. L., Schmutte, I. M., Simon, K. I., Weinberg, B. A., & Wing, C. (2020). *Determinants of disparities in covid-19 job losses*. National Bureau of Economic Research: Technical report.

[CR26] Queisser, M., Adema, W., Clarke, C. (2020). Covid-19, employment and women in oecd countries. CEPR VoxEu.org

[CR27] Sevilla A, Smith S (2020). Baby steps: The gender division of childcare during the covid-19 pandemic. Oxford Review of Economic Policy.

[CR28] Shibata I (2021). The distributional impact of recessions: The global financial crisis and the covid-19 pandemic recession. Journal of Economics and Business.

[CR29] Stantcheva, S. (2022). *Inequalities in the times of a pandemic*. National Bureau of Economic Research: Technical report.

[CR30] Zamarro G, Perez-Arce F, Prados MJ (2020). Gender differences in the impact of covid-19. KTLA.

[CR31] Zamarro G, Prados MJ (2021). Gender differences in couples’ division of childcare, work and mental health during covid-19. Review of Economics of the Household.

